# Reducing Anxiety and Risk: Views of the Supporting Dementia Caregivers After Death Advisory Board

**DOI:** 10.1093/geroni/igaf122.458

**Published:** 2025-12-31

**Authors:** Zachary Baker, Joahana Segundo, Ashley Millenbah

**Affiliations:** Arizona State University, Phoenix, Arizona, United States; Arizona State University, Phoenix, Arizona, United States; University of Minnesota, Minneapolis, Minnesota, United States

## Abstract

The Supporting Dementia Caregivers After Death Community Advisory Board (CAB) includes members with lived experience caring for people with dementia who have since died. Some CAB members expressed a strong fear of developing dementia, with a few stating they would prefer death over putting their families through caregiving. Many believed their own dementia risk was high. Given consensus that 14 lifestyle factors could potentially prevent 45% of dementia cases, we explored whether dementia-related anxiety could be leveraged to promote risk-reducing behaviors. CAB members were interested in learning about and improving these lifestyle factors, particularly social isolation, hearing loss, depression, and air pollution. While using dementia-related anxiety as motivation was not controversial, CAB members questioned whether dementia-related anxiety alone was a strong enough driver for behavior change. Instead, they suggested that discussions about dementia risk could serve as an entry point for broader conversations on prevention. Key themes emerged for designing interventions to improve lifestyle factors: (1) Timing—interventions soon after death might make risk feel more urgent but could be overwhelming during acute experiences of grief; (2) Self-Determination—interventions should empower individuals to choose which factors to address; (3) Leveraging existing resources—utilizing current programs rather than developing new ones; and (4) Perceived acuteness of risk—older adults may feel less able to modify risk, while younger adults may see the threat as too distant. These insights highlight the need for tailored, sensitive approaches to dementia risk reduction among bereaved dementia caregivers.

